# Hyperuricemia is Associated With 2- and 5-Year Adverse Outcomes in Patients With ST-Segment Elevation Myocardial Infarction Undergoing Percutaneous Coronary Intervention

**DOI:** 10.3389/fendo.2022.852247

**Published:** 2022-05-19

**Authors:** Xiao-Fang Tang, Chen He, Pei Zhu, Che Zhang, Ying Song, Jing-Jing Xu, Yi Yao, Na Xu, Ping Jiang, Lin Jiang, Zhan Gao, Xue-yan Zhao, Li-jian Gao, Lei Song, Yue-Jin Yang, Run-Lin Gao, Bo Xu, Jin-Qing Yuan

**Affiliations:** ^1^Department of Cardiology, Centre for Coronary Heart Disease, National Clinical Research Center for Cardiovascular Diseases, Fuwai Hospital, National Center for Cardiovascular Diseases, Chinese Academy of Medical Sciences and Peking Union Medical College, Beijing, China; ^2^Department of Cardiology, the Guangxi Zhuang Autonomous Region Workers’ Hospital, Nanning, China

**Keywords:** hyperuricemia, ST-segment myocardial infarction, percutaneous coronary intervention, long-term mortality, inflammatory response

## Abstract

**Background:**

Hyperuricemia has recently been identified as a risk factor of cardiovascular diseases; however, prognostic value of hyperuricemia in patients with ST-segment elevation myocardial infarction (STEMI) remained unclear. Simultaneously, the mechanism of this possible relationship has not been clarified. At present, some views believe that hyperuricemia may be related to the inflammatory response. Our study aimed to investigate the association between hyperuricemia and long-term poor prognosis and inflammation in STEMI patients undergoing percutaneous coronary intervention (PCI).

**Methods:**

A total of 1,448 consecutive patients with STEMI were studied throughout 2013 at a single center. The primary endpoint was all-cause death at 2- and 5-year follow-up. Inflammatory biomarkers were collected on admission of those patients: high sensitive C-reactive protein (hs-CRP), erythrocyte sedimentation rate (ESR), and white blood cell (WBC) count.

**Results:**

Hyperuricemia was associated with higher 2- and 5-year all-cause death in STEME patients compared to normouricemia (5.5% vs. 1.4%, P <0.001; 8.0% vs 3.9%, P = 0.004; respectively). After multivariable adjustment, hyperuricemia was still an independent predictor of 2-year all-cause death (hazard ratio (HR) =4.332, 95% confidence interval (CI): 1.990–9.430, P <0.001) and 5-year all-cause death (HR =2.063, 95% CI: 1.186–3.590, P =0.010). However, there was no difference in hs-CRP, ESR, and WBC count on admission in STEMI patients with hyperuricemia compared to normouricemia (P >0.05).

**Conclusions:**

Hyperuricemia was associated with higher risks of 2- and 5-year all-cause deaths in patients with STEMI undergoing PCI. However, this study did not find a correlation between hyperuricemia and inflammatory responses in newly admitted STEMI patients.

## Introduction

Acute myocardial infarction (AMI), including ST-segment elevation myocardial infarction (STEMI) and non-ST-segment elevation myocardial infarction (NSTEMI), is a major cause of morbidity and mortality worldwide. STEMI represents the most serious type of AMI and has a seriously adverse prognosis (1–3), which has the highest risk of intrahospital mortality (4–6). As we know, risk factors for STEMI patients, such as hypertension, dyslipidemia, diabetes mellitus, smoking, obesity, and so on, are common in clinical practice (7). Recently, hyperuricemia has gradually become a universally established cardiovascular risk factor (7–12).

Serum uric acid (SUA) is the final product of purine metabolism in humans, generated by xanthine oxidase or xanthine dehydrogenase (13). Hyperuricemia has been associated with many diseases such as cardiovascular diseases, stroke, chronic kidney disease, and hypertension (14). However, the link between hyperuricemia and poor prognosis in patients with STEMI remains controversial, with contradictory results from studies (15–18). Some studies have shown that the mortality of hyperuricemia was higher than that of normouricemia in patients with STEMI (18–23), although other studies have shown that hyperuricemia was not associated with high mortality in those patients (24, 25). Until now, the mechanism of this relationship has not been fully clarified. Studies have indicated that a high level of SUA could activate intracellular oxidative stress and inflammatory reactions (26), eventually leading to larger ischemic and infarct sizes in STEMI patients undergoing percutaneous coronary intervention (PCI) (27). This study investigates the association between hyperuricemia and long-term unfavorable prognosis and evaluates the relationship between hyperuricemia and the inflammatory response in STEMI patients undergoing PCI.

## Methods

### Study Population

This was a single-center prospective observational study. From 1 January 2013, to 31 December 2013, a total of 1,448 consecutive patients with STEMI undergoing PCI were enrolled at the Fuwai Hospital in Beijing, China. STEMI was diagnosed according to the guidelines published by the American College of Cardiology (28). All patients were given standard treatment for STEMI. The study protocol was approved by the ethics committee of our hospital, and all patients signed written informed consent before study entry.

Routine blood samples were collected from each patient with STEMI upon study admission, namely, SUA, serum creatinine, high sensitive C-reactive protein (hs-CRP), erythrocyte sedimentation rate (ESR), white blood cell (WBC) count, N-Terminal pro-brain natriuretic peptide (NT-proBNP), and so on. Hyperuricemia was diagnosed as SUA levels greater than 420 mmol/L (men) and 360 mmol/L (women). Based on SUA levels, 275 patients were divided into the hyperuricemia group and 1,173 patients were divided into normouricemia group. Independent clinical research coordinators gathered clinical and procedural data in a database.

### Procedural and Medications

The choice of treatment strategy for PCI, including the type of stent, was left to the discretion of the treating physicians. STEMI patients scheduled for PCI received a loading dose of 300 mg aspirin and a loading dose of the P2Y12 inhibitors (a dose of 180 mg ticagrelor or a dose of 300 mg or 600 mg clopidogrel) as soon as possible. Then, aspirin was advised at a dose of 75–100 mg daily for life; clopidogrel 75 mg daily or ticagrelor 90 mg twice daily was prescribed for at least 1 year.

### Endpoints and Definitions

All the patients were assessed by clinic or phone at 1, 3, 6, and 12 months after PCI, and annually thereafter until 5 years. The primary endpoint was all-cause death. Secondary endpoints included cardiac death, major adverse cardiac, and cerebrovascular events (MACCE). The diagnostic criterion for MI was based on the Third Universal Definition of myocardial infarction (29). All deaths were considered cardiac unless an unequivocal noncardiac cause could be established. MACCE was the composite of all-cause death, MI, unplanned revascularization, or stroke. Two independent physicians adjudicated the events after reviewing the original documents, which were blinded to the data.

### Statistical Analysis

Continuous variables were expressed as mean ± standard deviation, and Student’s t-test or the Mann–Whitney U test was used for comparison between two groups. Numbers and percentages were used to present categorical variables, and the chi-square test or Fisher’s exact test was used for comparison between two groups, as appropriate. The Kaplan–Meier method was used to calculate the event-free survival rates and the log-rank test to compare them. Confirm the association between the different groups and each outcome of interest using the univariable and multivariable Cox proportional regression models. Multivariable Cox regression analysis was used to identify associations between two groups and each outcome of interest after adjusting for potential confounding factors, namely, age, gender, hypertension, hyperlipidemia, diabetes mellitus (DM), chronic kidney disease (CKD), current smoker, the family history of coronary heart disease (CHD), left ventricular ejection fraction (LVEF <40 or ≥40), SYNTAX score, old myocardial infarction (OMI), previous PCI, previous coronary artery bypass graft (CABG), and inflammatory markers, namely, hs-CRP, ESR, and WBC counts. All P-values were two-sided, and P <0.05 was considered statistically significant. All statistical analyses were performed using SPSS software version 22.0 (IBM Corp., Armonk, NY, USA).

## Results

**Figure 1:**
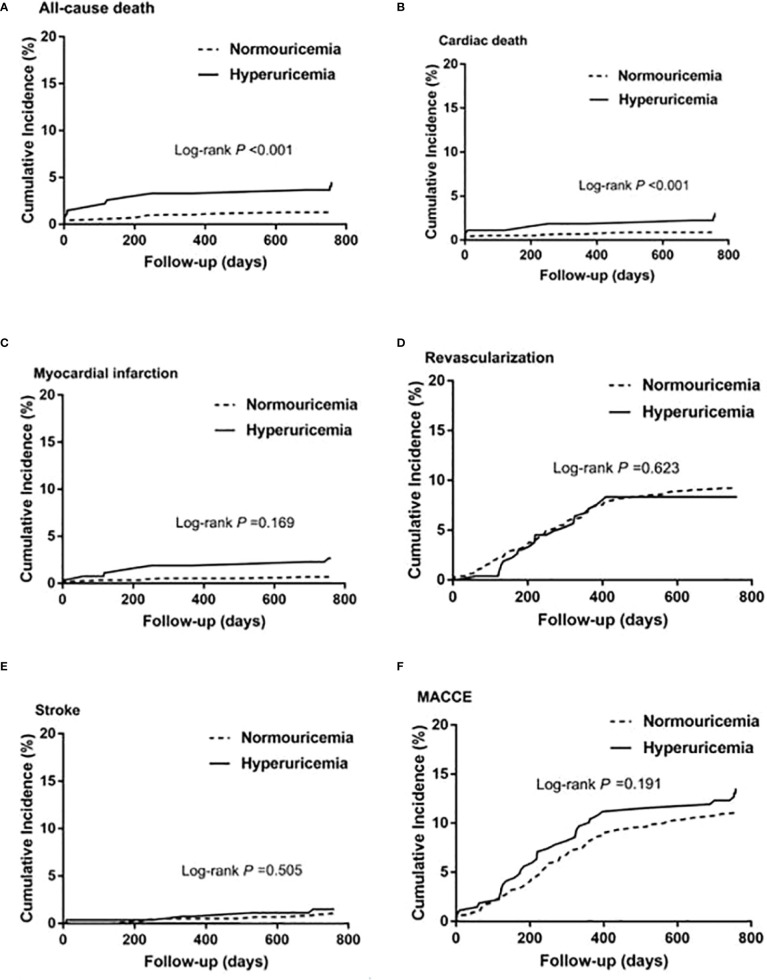
Kaplan–Meier survival curves for 2-year primary and secondary outcomes in STEMI patients with PCI. **(A)** All-cause death, **(B)** cardiac death, **(C)** myocardial infarction, **(D)** unplanned revascularization, **(E)** stroke, and **(F)** MACCE. MACCE, major adverse cardiac and cerebrovascular events.

**Figure 2:**
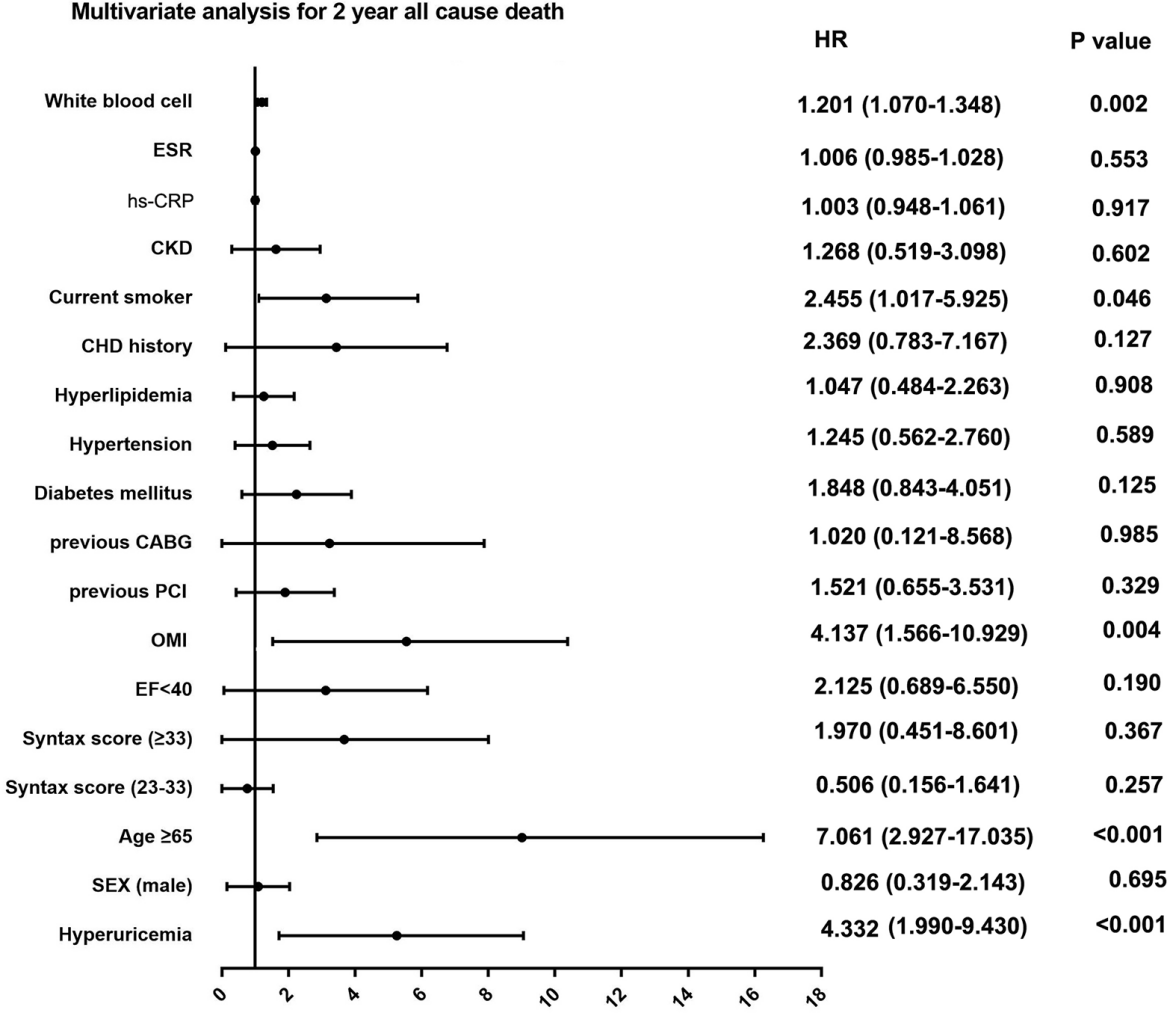
Multivariate analysis for 2-year primary outcome in patients with STEMI. ESR, erythrocyte sedimentation rate; hs-CRP, high sensitive C-reactive protein; CKD, chronic kidney disease; CHD, coronary heart disease; CABG, coronary artery bypass grafting; PCI, percutaneous coronary intervention; OMI, old myocardial infarction; EF, ejection fraction.

Among patients with STEMI (n = 1,448), 275 (19.0%) had hyperuricemia and 1,173 (81%) had normouricemia. The clinical baseline and procedural characteristics data are shown in **Table 1**. STEMI patients with hyperuricemia were more likely to be male and had higher BMI, serum creatinine, and lower creatinine clearance than those with normouricemia (P <0.05). The rates of hypertension, CKD, and LVEF <40% were also higher in STEMI patients with hyperuricemic than in those with normouricemia (P <0.05). There were no differences in hs-CRP, ESR, WBC counts, and NT-ProBNP between hyperuricemic and normouricemic patients on admission (P >0.05). During hospitalization and follow-up, only 14 patients (5.1%) with hyperuricemia were on urate-lowering drugs (xanthine oxidase inhibitors). During the 2-year follow-up after PCI, STEMI patients with hyperuricemia had significantly higher all-cause and cardiac deaths than those patients with normouricemia (5.5% vs. 1.4%, P <0.001; and 4.0% vs. 0.9%, P = 0.001; **Table 2**). The rates of MI, unplanned revascularization, stroke, and MACCE were not significantly different between STEMI patients with hyperuricemia and normouricemia (P >0.05). Cumulative survival rate curves for the primary and secondary endpoints of 2-year follow-up are shown in **Figure 1** by Kaplan–Meier analysis. Hyperuricemia was significantly associated with increased risks of all-cause death (HR = 4.081, 95% CI: 2.018–8.255, P <0.001) and cardiac death (HR = 4.341, 95% CI: 1.882–10.012, P = 0.001) in STEMI patients with PCI by univariate analyses (**Table 2**). After multivariable analysis adjusting for baseline characteristics and inflammatory markers, hyperuricemia was still significantly associated with increased risks of all-cause death (HR = 4.332, 95% CI: 1.990–9.430, P <0.001) and cardiac death (HR = 4.635, 95% CI: 1.872–11.476, P = 0.001) in STEMI patients with PCI (**Table 2**). For clinical endpoints that were measured, age older than 65, WBC counts, current smoker, and a history of OMI were found to be the independent predictors of all-cause death in STEMI patients with PCI (**Figure 2**).

**Table 1:** Baseline characteristics of the study population.

Variable	STEMI		p-value
	Hyperuricemia	Normouricemia
	(n = 275)	(n = 1173)
Age ≥65 (n, %)	66 (24.0)	250 (21.3)	0.332
Sex (male, n, %)	220 (80)	1,018 (86.8)	0.004
Body mass index (kg/m^2^)	26.8 ± 3.4	25.8 ± 3.1	<0.001
LVEF <40% (n, %)	16 (5.9)	29 (2.5)	0.004
Creatinine clearance (ml/min)	84.0 ± 20.9	92.7 ± 15.8	<0.001
hs-CRP (mg/L)	5.5 ± 4.8	6.2 ± 5.1	0.115
ESR (mm/h)	15.7 ± 15.4	15.5 ± 15.5	0.810
White blood cell count (10^9^/L)	7.47 ± 2.29	7.58 ± 2.34	0.489
NT-ProBNP (pmol/l)	1,195.5 ± 921.3	1,103.5 ± 767.5	0.202
HbA1c (%)	6.4 ± 1.1	6.7 ± 1.5	0.184
Serum uric acid (mmol/L)	470.7 ± 58.9	312.5 ± 60.8	<0.001
Chronic kidney disease (n, %)	38 (13.8)	44 (3.8)	<0.001
Hypertension (n, %)	169 (61.5)	631 (53.8)	0.021
Hyperlipidemia (n, %)	179 (65.1)	692 (59.0)	0.063
Diabetes mellitus (n, %)	55 (20.0)	322 (27.5)	0.011
Current smoker (n, %)	187 (68.0)	803 (68.5)	0.883
Family history of CHD (n, %)	68 (24.7)	287 (24.5)	0.928
Stroke history (n, %)	31 (11.3)	99 (8.4)	0.139
Peripheral artery disease (n, %)	1 (0.4)	13 (1.1)	0.490
Old myocardial infarction (n, %)	19 (6.9)	74 (6.3)	0.715
Previous PCI (n, %)	72 (26.2)	270 (23.0)	0.266
Previous CABG (n, %)	9 (3.3)	12 (1.0)	0.01
Medication (n, %)			
Aspirin	268 (97.5)	1,151 (98.1)	0.475
Clopidogrel	268 (97.5)	1,149 (98.0)	0.607
DAPT	264 (96.0)	1,138 (97.0)	0.387
Statin	261 (94.9)	1,112 (94.8)	0.941
B-blocker	252 (91.6)	1,062 (90.5)	0.571
Lesions involving LM (n, %)	6 (2.2)	18 (1.5)	0.434
Lesions involving LAD (n, %)	247 (89.8)	1,068 (91.0)	0.525
Lesions involving LCX (n, %)	42 (15.3)	185 (15.8)	0.838
Lesions involving RCA (n, %)	18 (6.5)	96 (8.2)	0.364
Single lesion (n, %)	70 (25.5)	317 (27.0)	0.596
Double lesions (n, %)	89 (32.4)	375 (32.0)	0.900
Triple lesions (n, %)	116 (42.2)	481 (41.0)	0.721
SYNTAX score			
≤22	229 (83.3)	1,033 (88.1)	0.033
23–32	36 (13.1)	118 (10.1)	0.142
≥33	10 (3.6)	22 (1.9)	0.074

STEMI, ST-segment elevation myocardial infarction; LVEF, left ventricular ejection fraction; hs-CRP, high sensitive-C reaction protein; ESR, erythrocyte sedimentation rate; NT-ProBNP, N-Terminal pro-brain natriuretic peptide; HbA1c, glycosylated hemoglobin, type A1C; CHD, coronary heart disease; PCI, Percutaneous coronary intervention; CABG, Coronary artery bypass graft; DAPT, dual antiplatelet treatment; LM, left main disease; LAD, left anterior descending; LCX, left circumflex; RCA, right coronary artery; SYNTAX, the synergy between percutaneous coronary intervention with Taxus and cardiac surgery.

**Table 2:** Risks of 2-year primary and secondary outcomes in patients with STEMI.

Outcomes	No. (%)	Hazard ratio		Adjusted hazard ratio	Adjusted
		(95% CI)	*P*	(95% CI)	*P*
**Primary outcomes**					
All-cause death					
Hyperuricemia	15 (5.5)	4.081 (2.018–8.255)	<0.001	4.332 (1.990–9.430)	<0.001
Normouricemia	16 (1.4)	Reference		Reference	
**Secondary outcomes**					
Cardiac death					
Hyperuricemia	11 (4.0)	4.341(1.882–10.012)	0.001	4.635 (1.872–11.476)	0.001
Normouricemia	11 (0.9)	Reference		Reference	
Myocardial infarction					
Hyperuricemia	10 (3.6)	1.655 (0.798–3.432)	0.176	1.305 (0.600–2.839)	0.502
Normouricemia	26 (2.2)	Reference		Reference	
Unplanned revascularization					
Hyperuricemia	22 (8.0)	0.891 (0.564–1.410)	0.623	0.870 (0.543–1.393)	0.561
Normouricemia	108 (9.2)	Reference		Reference	
Stroke					
Hyperuricemia	4 (1.5)	1.466 (0.473–4.547)	0.507	1.366 (0.420–4.438)	0.604
Normouricemia	12 (1.0)	Reference		Reference	
MACCE					
Hyperuricemia	43 (15.6)	1.253 (0.893–1.759)	0.192	1.152 (0.811–1.638)	0.429
Normouricemia	149 (12.7)	Reference		Reference	

CI, confidence interval; MACCE, major adverse cardiac and cerebrovascular events.

**Figure 3:**
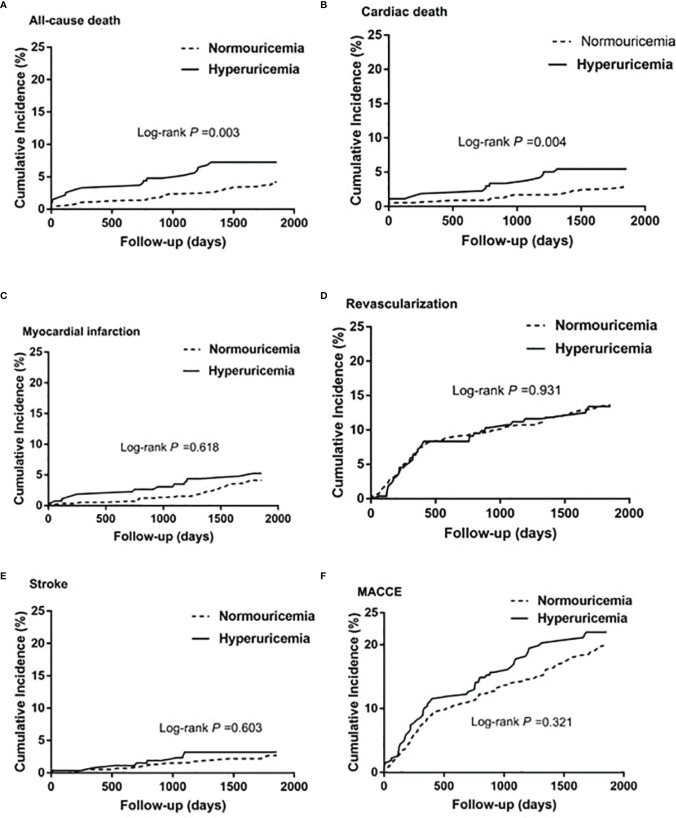
Kaplan–Meier survival curves for 5-year primary and secondary outcomes in STEMI patients with PCI. **(A)** All-cause death, **(B)** cardiac death, **(C)** myocardial infarction, **(D)** unplanned revascularization, **(E)** stroke, and **(F)** MACCE. MACCE, major adverse cardiac and cerebrovascular events.

**Figure 4:**
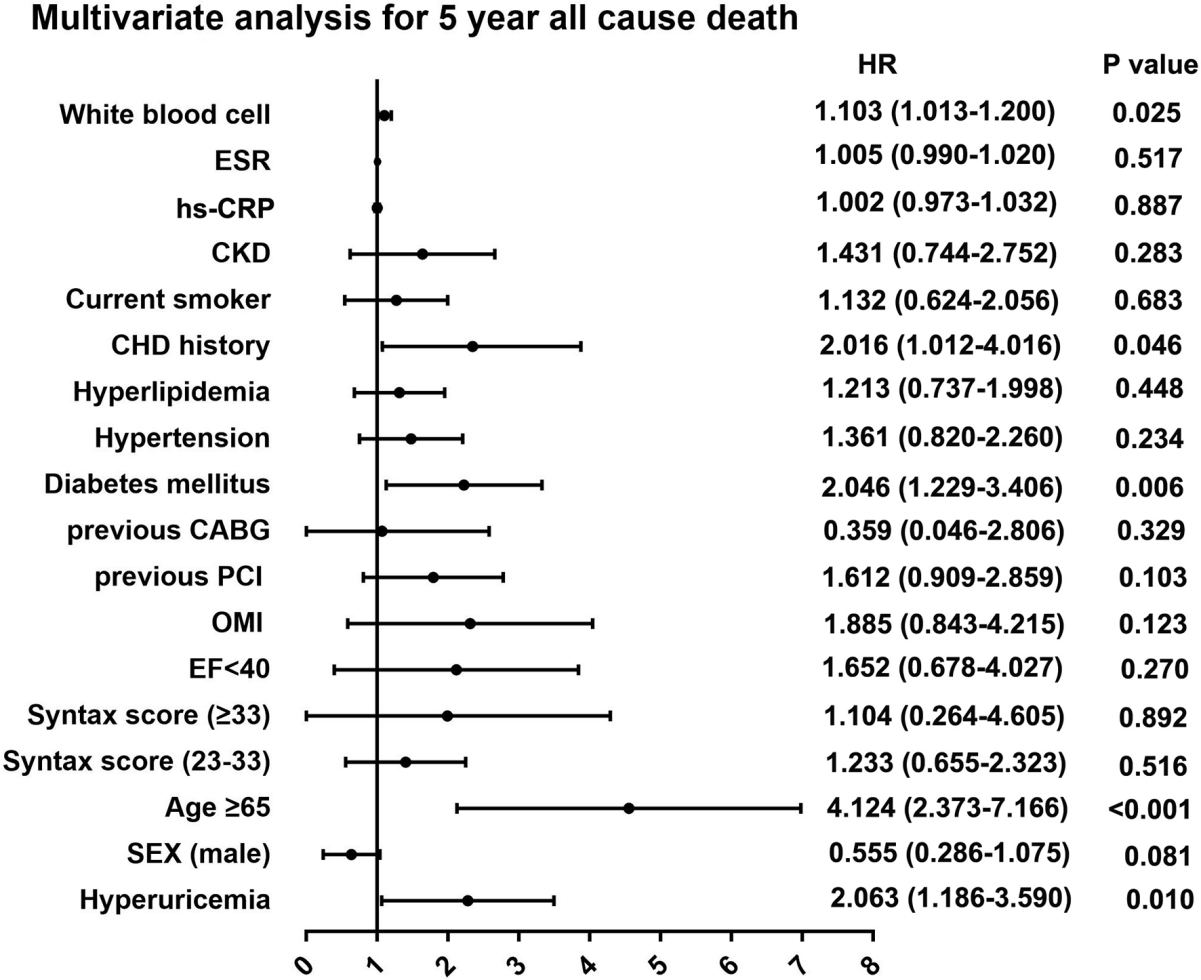
Multivariate analysis for 5-year primary outcome in patients with STEMI. ESR, erythrocyte sedimentation rate; hs-CRP, high sensitive C-reactive protein; CKD, chronic kidney disease; CHD, coronary heart disease; CABG, coronary artery bypass grafting; PCI, percutaneous coronary intervention; OMI, old myocardial infarction; EF, ejection fraction.

During the 5-year follow-up after PCI, the rates of all-cause and cardiac deaths were also significantly higher in STEMI patients with hyperuricemia than in normouricemia (8.0% vs. 3.9%, P = 0.004; 6.2% vs. 2.8%, P = 0.006; **Table 3**). The rates of MI, unplanned revascularization, stroke, and MACCE were not significantly different between STEMI patients with hyperuricemia and normouricemia (p >0.05). Cumulative survival rate curves for the primary and secondary endpoints of the 5-year follow-up are shown in **Figure 3** by Kaplan–Meier analysis. Hyperuricemia was also significantly associated with increased risks of all-cause death (HR = 2.118, 95% CI: 1.274–3.520, P = 0.004) and cardiac death (HR = 2.282, 95% CI: 1.271–4.097, P = 0.006) in STEMI patients treated with PCI by univariate analyses (**Table 3**). After multivariable analysis adjusting for baseline characteristics and inflammatory markers, hyperuricemia was also significantly associated with increased risks of all-cause death (HR = 2.063, 95% CI: 1.186–3.590, P = 0.010) and cardiac death (HR = 2.153, 95% CI: 1.142–4.058, P = 0.018) in STEMI patients treated with PCI (**Table 3**). For clinical endpoints that were measured, gender for age older than 65, CHD history, WBC counts, and DM were found to be independent predictors of all-cause death in patients with STEMI during the 5-year follow-up (**Figure 4**).

**Table 3:** Risks of 5-year primary and secondary outcomes in patients with STEMI.

Outcomes	No. (%)	Hazard ratio		Adjusted hazard ratio	Adjusted
		(95% CI)	*P*	(95% CI)	*P*
**Primary outcomes**					
All-cause death					
Hyperuricemia	22 (8.0)	2.118 (1.274–3.520)	0.004	2.063 (1.186–3.590)	0.010
Normouricemia	46 (3.9)	Reference		Reference	
**Secondary outcomes**					
Cardiac death					
Hyperuricemia	17 (6.2)	2.282 (1.271–4.097)	0.006	2.153 (1.142–4.058)	0.018
Normouricemia	33 (2.8)	Reference		Reference	
Myocardial infarction					
Hyperuricemia	16 (5.8)	1.150 (0.663–1.994)	0.619	0.926 (0.516–1.661)	0.796
Normouricemia	61 (5.2)	Reference		Reference	
Unplanned revascularization					
Hyperuricemia	34 (12.4)	0.984 (0.678–1.427)	0.931	0.986 (0.672–1.446)	0.941
Normouricemia	152 (13.0)	Reference		Reference	
Stroke					
Hyperuricemia	8 (2.9)	1.230 (0.562–2.691)	0.604	1.071 (0.477–2.408)	0.868
Normouricemia	29 (2.5)	Reference		Reference	
MACCE					
Hyperuricemia	63 (22.9)	1.151 (0.872–1.519)	0.321	1.089 (0.816–1.452)	0.563
Normouricemia	240 (20.5)	Reference		Reference	

CI, confidence interval; MACCE, major adverse cardiac and cerebrovascular events.

## Discussion

This study found an association between hyperuricemia and adverse clinical outcomes in STEMI patients undergoing PCI. The main findings were that hyperuricemia was an independent risk factor for increased risk of all-cause death and cardiac death in STEMI patients undergoing PCI before and after multivariable analyses during long-term follow-up. Meanwhile, age and WBC count were also independent risk predictors of all-cause death in STEMI patients undergoing PCI. However, this study did not find an association between hyperuricemia and inflammatory responses in STEMI patients on admission.

Previous studies had explored the association of hyperuricemia with mortality in STEMI patients, but the results were controversial. Kaya et al. found that STEMI patients with hyperuricemia were associated with higher in-hospital and long-term mortality (15). Levantesi et al. found that hyperuricemia significantly increased the risk of all-cause and cardiovascular death in STEMI patients with long-term follow-up (16). Although Lazzeri et al. found that STEMI patients with hyperuricemia had a higher risk of in-hospital mortality than those with normouricemia (18), another study failed to demonstrate the association of hyperuricemia with mortality in STEMI patients (24): although hyperuricemia had a higher risk of mortality in 856 STEMI patients, the significance disappeared after adjustment for renal function (24). Our study found that hyperuricemia was associated with higher 2-year and 5-year all-cause deaths and cardiac deaths in patients with STEMI undergoing PCI. Meanwhile, only 14 patients (5.1%) with hyperuricemia in this cohort were taking urate-lowering drugs (xanthine oxidase inhibitors). It can be seen that in clinical practice, the management of hyperuricemia is not active in China. In future studies, the clinical benefit of uric acid-lowering therapy in STEMI patients with hyperuricemia can be further explored.In our study, STEMI patients with hyperuricemia had a higher proportion of obesity (higher BMI), hypertension, CKD, and lower LVEF, which may be important factors leading to more severe myocardial damage. However, after adjustment for age, gender, BMI, SYNTAX score, LVEF, OMI, the history of previous PCI, and previous CABG, DM, hypertension, hyperlipidemia, current smoker, the family history of CHD, CKD, and inflammatory markers, hyperuricemia was still an independent risk predictor of all-cause death and cardiac death in STEMI patients undergoing PCI during long-term follow-up. Meanwhile, age, DM, and WBC counts were also independent predictors of all-cause death in STEMI patients undergoing PCI.

The pathogenesis of hyperuricemia associated with adverse clinical outcomes in patients with STEMI remains undetermined. Uric acid is the final metabolite of purine catalyzed by xanthine oxidase (30). Xanthine oxidase activity and UA production lead to the generation of oxygen free radicals and may be involved in the atherosclerosis process caused by oxygen free radicals (31). During tissue ischemia, the enzymatic effect of xantine oxidase is the production of reactive oxygen species (ROS) and uric acid in experimental models (32, 33); hyperuricemia *per se* has been described to impair endothelium-dependent vasodilatation by reduction of NO-synthase in animal experiments (34). Preclinical studies have found that oxidative stress and inflammatory response can be triggered by elevatedSUA (35–38). Mandurino-Mirizzi et al. found that intracellular oxidative stress and inflammatory responses might be triggered by elevated SUA, which may contribute to ischemic/reperfusion injury, coronary microvascular obstruction, and larger infarct size during revascularization and reperfusion in STEMI patients (27).

Previous studies reported that SUA was correlated with CRP in ACS patients (17, 39). Magnoni et al. found SUA had a positive association with CRP in ACS patients who died during hospitalization. However, there was no association between SUA and CRP in ACS patients who survived discharge (17). Recently, a small study found a significant linear correlation between SUA levels and hs-CRP peak values in STEMI patients with PCI, which suggested that elevated SUA had a possible association with a greater inflammatory response in this setting (26, 40). However, in our study, we did not find any correlations between hyperuricemia and inflammatory response markers (WBC counts, ESR, and hs-CRP) in STEMI patients undergoing PCI on admission. Until now, the mechanism by which hyperuricemia was associated with poor prognosis in STEMI patients undergoing PCI remained unclear. In future studies, we intend to closely monitor changes in uric acid and inflammatory markers throughout the pathogenesis of STEMI patients to explore the relationship between hyperuricemia and inflammatory response.

### Limitations

This study has several limitations. First, this study was based on a real-world observational study and may therefore be subject to residual confounders. Multivariate analysis was used in this study, but some unmeasured confounding factors could not be ruled out that might have influenced the findings. Hence, the results of this study should be interpreted with caution because of the existence of selection bias or confounding bias that cannot be excluded. Second, this study was a single-center study with all participants from one center, which may limit the generalizability of the results. Third, uric acid and inflammatory markers monitored on admission do not reflect the overall course of STEMI patients undergoing PCI. In future studies, we intend to monitor uric acid and inflammatory markers throughout the pathogenesis of STEMI and explore the relationship between hyperuricemia and inflammatory responses.

### Conclusions

This study suggests that hyperuricemia was an independent risk factor for all-cause and cardiac deaths in STEMI patients undergoing PCI during long-term follow-up and that it is a commonly used biomarker for long-term prognostic stratification in STEMI patients undergoing PCI. However, the underlying pathophysiological mechanisms must be further explored in those patients with hyperuricemia.

## Data Availability Statement

The raw data supporting the conclusions of this article will be made available by the authors, without undue reservation.

## Ethics Statement

The studies involving human participants were reviewed and approved by the ethics committee of Fuwai Hospital. The patients/participants provided their written informed consent to participate in this study.

## Author Contributions

Y-QY, BX, and X-FT contributed to conception and design of the study. Y-QY, BX, X-FT, R-LG, Y-JY, LS, L-JG, X-yZ, and ZG contributed to performing experiments and acquisition of data. X-FT, CH, PZ, CZ, YS, J-JX, YY, NX, PJ, and LJ contributed to analysis and interpretation of data. YQY, BX, XFT, RLG, and YJY contributed to drafting the article or revising it critically for important intellectual content. All authors listed have made a substantial, direct, and intellectual contribution to the work and approved it for publication.

## Funding

This study was supported by the National Science and Technology Support Program of China and sub-project (Nos. 2016YFC1301300 and 2016YFC1301301), the National Clinical Research Center for Cardiovascular Diseases, Fuwai Hospital, Chinese Academy of Medical Sciences (CAMS) (Grant No.NCRC2020013), and the CAMS Innovation Fund for Medical Sciences (CIFMS): 2020-I2M-C&T-B-049.

## Conflict of Interest

The authors declare that the research was conducted in the absence of any commercial or financial relationships that could be construed as a potential conflict of interest.

## Publisher’s Note

All claims expressed in this article are solely those of the authors and do not necessarily represent those of their affiliated organizations, or those of the publisher, the editors and the reviewers. Any product that may be evaluated in this article, or claim that may be made by its manufacturer, is not guaranteed or endorsed by the publisher.
