# Supplementary material for: Hyperuricemia is Associated With 2- and 5-Year Adverse Outcomes in Patients With ST-Segment Elevation Myocardial Infarction Undergoing Percutaneous Coronary Intervention
Source: Front Endocrinol (Lausanne). 2022 May 19;13:852247. doi: 10.3389/fendo.2022.852247 (PMC9160184; doi:10.3389/fendo.2022.852247)
Supplement: Supplementary file 1 [file Table_1.docx]

**Supplementary Table 1. Uric acid and inflammatory markers of the study population during follow-up**

| Variable | STEMI | |  |
| --- | --- | --- | --- |
|  | Hyperuricemia | Normouricemia |  |
|  | (n =141) | (n =652) | p value |
| Serum uric acid (mmol/L) | 423.6±84.5 | 337.1±72.0 | <0.001 |
| hs-CRP (mg/L) | 2.55±3.15 | 1.68±2.16 | 0.002 |
| ESR (mm/h) | 9.2 ± 12.2 | 7.2 ± 9.5 | 0.663 |
| White blood cell count (10^9/L) | 6.85 ± 1.62 | 6.77 ± 1.64 | 0.596 |

**Supplementary Table 2.Multivariable Cox proportional hazards model for 2-year cardiac death.**

|  | HR (95% CI) | *P*-value |
| --- | --- | --- |
| Uric acid, mmol/L | 1.008 (1.004‒1.014) | <0.001 |
| Male sex | 0.749 (0.226-2.489) | 0.638 |
| Age (≥65 vs. <65) | 5.246 (1.873-14.693) | 0.002 |
| Previous MI | 5.079 (1.786‒14.445) | 0.005 |
| Previous PCI | 2.205 (0.797-6.103) | 0.128 |
| Previous CABG | 1.323 (0.150-11.690) | 0.801 |
| Hypertension | 1.213 (0.473‒3.112) | 0.688 |
| Diabetes | 1.766 (0.663‒4.699) | 0.255 |
| Hyperlipidemia | 1.001 (0.395-2.539) | 0.998 |
| CHD history | 4.026 (0.919‒13.703) | 0.060 |
| Ejection fraction (<40 vs. ≥40) | 1.503 (0.320‒7.070) | 0.606 |
| CKD | 1.266 (0.408‒3.927) | 0.683 |
| Current smoker | 4.139 (1.412‒12.130) | 0.010 |
| HSCRP, mg/L | 0.975 (0.880-1.080) | 0.626 |
| ESR, mm/h | 0.993 (0.958-1.030) | 0.720 |
| White blood cell counts, 10^9/L | 1.213 (1.056-1.393) | 0.006 |
| SYNTAX score |  | 0.299 |
| 23‒32 vs ≤22 | 0.460 (0.095‒2.229) | 0.335 |
| ≥33 vs ≤22 | 2.385 (0.513‒11.092) | 0.268 |

**Supplementary Table 3.Multivariable Cox proportional hazards model for 5 year cardiac death.**

|  | HR (95% CI) | *P*-value |
| --- | --- | --- |
| Uric acid, mmol/L | 1.004 (1.001‒1.007) | 0.007 |
| Male sex | 0.642 (0.288-1.431) | 0.279 |
| Age (≥65 vs. <65) | 3.446 (1.809-6.564) | <0.001 |
| Previous MI | 2.472 (1.065‒5.737) | 0.035 |
| Previous PCI | 1.577 (0.802-3.101) | 0.187 |
| Previous CABG | 0.557 (0.069-4.490) | 0.583 |
| Hypertension | 1.206 (0.664‒2.192) | 0.539 |
| Diabetes | 2.052 (1.135‒3.708) | 0.017 |
| Hyperlipidemia | 1.256 (0.700-2.254) | 0.445 |
| CHD history | 2.080 (0.921‒4.694) | 0.078 |
| Ejection fraction (<40 vs. ≥40) | 1.277 (0.431‒3.778) | 0.659 |
| CKD | 1.805 (0.833‒3.910) | 0.134 |
| Current smoker | 1.320 (0.659‒2.643) | 0.433 |
| HSCRP, mg/L | 1.000 (0.964-1.038) | 0.982 |
| ESR, mm/h | 1.003 (0.984-1.021) | 0.778 |
| White blood cell counts, 10^9/L | 1.111 (1.008-1.225) | 0.035 |
| SYNTAX score |  | 0.927 |
| 23‒32 vs ≤22 | 1.032 (0.469‒2.271) | 0.938 |
| ≥33 vs ≤22 | 1.331 (0.313‒5.656) | 0.698 |

**Supplementary Table 4. Number of missing values and corresponding dispositions.**

|  | No. of Missing value | Disposition |
| --- | --- | --- |
| Glycosylated hemoglobin, type A1C | 36 (2.5%) | Median imputation |
| Left ventricular ejection fraction | 13 (0.9%) | Median imputation |
| LVEF <40% | 13 (0.9%) | Mode imputation |
| high sensitive-C reaction protein | 29 (2.0%) | Median imputation |
| Erythrocyte sedimentation rate | 21 (1.5%) | Median imputation |
| White blood cell count | 19 (1.3%) | Median imputation |
| N-Terminal pro-brain natriuretic peptide | 39 (2.7%) | Median imputation |
